# Mesenchymal Stem Cells in Oriented PLGA/ACECM Composite Scaffolds Enhance Structure-Specific Regeneration of Hyaline Cartilage in a Rabbit Model

**DOI:** 10.1155/2018/6542198

**Published:** 2018-02-13

**Authors:** Weimin Guo, Xifu Zheng, Weiguo Zhang, Mingxue Chen, Zhenyong Wang, Chunxiang Hao, Jingxiang Huang, Zhiguo Yuan, Yu Zhang, Mingjie Wang, Jiang Peng, Aiyuan Wang, Yu Wang, Xiang Sui, Wenjing Xu, Shuyun Liu, Shibi Lu, Quanyi Guo

**Affiliations:** ^1^Institute of Orthopaedics, Chinese PLA General Hospital, Beijing Key Lab of Regenerative Medicine in Orthopaedics, Key Laboratory of Musculoskeletal Trauma & War Injuries, PLA, No. 28 Fuxing Road, Haidian District, Beijing 100853, China; ^2^Department of Orthopedic Surgery, First Affiliated Hospital, Dalian Medical University, No. 222 Zhongshan Road, Xigang District, Dalian 116011, China; ^3^First Department of Orthopedics, First Affiliated Hospital of Jiamusi University, No. 348 Dexiang Road, Xiangyang District, Jiamusi 154003, China; ^4^Institute of Anesthesiology, Chinese PLA General Hospital, No. 28 Fuxing Road, Haidian District, Beijing 100853, China

## Abstract

Articular cartilage lacks a blood supply and nerves. Hence, articular cartilage regeneration remains a major challenge in orthopedics. Decellularized extracellular matrix- (ECM-) based strategies have recently received particular attention. The structure of native cartilage exhibits complex zonal heterogeneity. Specifically, the development of a tissue-engineered scaffold mimicking the aligned structure of native cartilage would be of great utility in terms of cartilage regeneration. Previously, we fabricated oriented PLGA/ACECM (natural, nanofibrous, articular cartilage ECM) composite scaffolds. In vitro, we found that the scaffolds not only guided seeded cells to proliferate in an aligned manner but also exhibited high biomechanical strength. To detect whether oriented cartilage regeneration was possible in vivo, we used mesenchymal stem cell (MSC)/scaffold constructs to repair cartilage defects. The results showed that cartilage defects could be completely regenerated. Histologically, these became filled with hyaline cartilage and subchondral bone. Moreover, the aligned structure of cartilage was regenerated and was similar to that of native tissue. In conclusion, the MSC/scaffold constructs enhanced the structure-specific regeneration of hyaline cartilage in a rabbit model and may be a promising treatment strategy for the repair of human cartilage defects.

## 1. Introduction

Articular cartilage defects are very common due to the increasing numbers of patients with traumatic injuries and osteoarthritis (OA) [1]. Cartilage naturally lacks a blood supply and nerves [2]. Hence, the repair of cartilage lesions is very difficult due to the poor healing capacity. Current clinical approaches to cartilage repair are limited [3]. In contrast to traditional treatments, such as subchondral drilling and microfracture, tissue engineering is becoming a very promising alternative treatment for cartilage injuries [4].

The scaffold, the seed cells, and the nature of biochemical or biomechanical stimulation are the three critical elements of tissue engineering and regeneration medicine [5, 6]. Natural and synthetic polymers have been investigated widely as scaffold materials for cartilage tissue engineering [7]. Most natural polymers preserve or mimic the essential biochemical components of native tissues [8]. Surface attachment of specific signal peptides may enhance cell attachment, proliferation, and redifferentiation [9]. Hence, bioactivity is always better than that afforded by synthetic polymers. Decellularized extracellular matrix (ECM) is one the most popular biomaterials used for cartilage regeneration [10–12]. However, the mechanical properties of such scaffolds are inadequate. Synthetic polymers exhibit good biomechanical properties, satisfactory biocompatibility, and a controllable biodegradation rate [13]. However, most synthetic polymers are hydrophobic and lack intrinsic bioactivity; thus, they do not facilitate cell seeding or stem cell chondrogenesis [14].

Biomimetic structures similar to native tissue are of great utility in scaffold fabrication [15–19]. Articular cartilage exhibits zonal distinctions in cell distribution and collagen fiber structure. Notably, the structural alignment of deep cartilage tissue runs vertical to that of subchondral bone [20]. Some studies have shown that scaffold alignment may have major effects on the orientation of cartilage regeneration [21, 22].

To combine the advantages afforded by synthetic and natural polymeric materials, we previously developed oriented PLGA/ACECM composite scaffolds using an improved, thermally induced phase-separation approach [21, 23]. We confirmed that the composite scaffolds not only exhibited good affinities for cells and biomechanical capacities but also featured the formation of cartilage that was structurally aligned.

Chondrocytes are the (uniquely) functional cells of articular cartilage [24]. However, chondrocyte harvesting is traumatic, and adequate in vitro amplification of cells is difficult. Stem cell-based regeneration strategies play critical roles in cartilage engineering [25]. Mesenchymal stem cells (MSCs) have the advantages of easy harvesting, a minimal requirement for bodily invasion, relatively good proliferation abilities, and the capacity to engage in chondrogenesis in vitro [26–28]. However, no in vivo study of cartilage regeneration using MSCs loaded into oriented PLGA/ACECM composite scaffolds has yet appeared.

**Figure 1:**
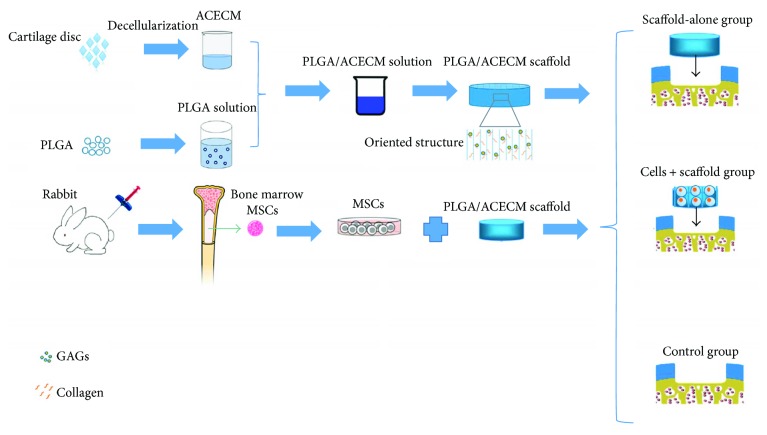
Schematic illustration of the experimental design. Autologous bone marrow-derived MSCs were loaded into oriented PLGA/ACECM composite scaffolds and implanted to regenerate cartilage defects in a rabbit model. The study involved three groups: a cell-loaded scaffold group, a scaffold-alone group, and an untreated (control) group.

In the present study, we first isolated autologous bone marrow-derived MSCs and seeded these cells into oriented PLGA/ACECM composite scaffolds. We then implanted the cell-scaffold constructs into cartilage defects created in rabbits (Figure 1). We found that the cell-scaffold constructs facilitated cartilage regeneration; aligned regeneration was evident by 24 weeks postimplantation.

## 2. Materials and Methods

### 2.1. Isolation and Culture of MSCs

After the work was approved by the Animal Care and Experimental Committee of the Chinese People's Liberation Army General Hospital, bone marrow-derived MSCs were isolated from anesthetized mature New Zealand white rabbits (weight 2.5–3.0 kg) and cultured as described previously [29]. Briefly, 8 mL amounts of bone marrow aspirate were harvested from the iliac crest, followed by Lymphoprep gradient centrifugation at 2000 rpm for 25 min at room temperature. The mononuclear cells were separated and washed twice in Hank's solution. These cells were resuspended in a regular growth medium with a low glucose level (Dulbecco's modified Eagle's medium-low glucose (DMEM-LG); Sigma-Aldrich, USA) supplemented with 10% (*v*/*v*) fetal bovine serum (FBS, Gibco, USA) and then plated at a density of 1 × 10^6^ cells/cm^2^ in a humidified atmosphere under 5% (*v*/*v*) CO_2_ at 37°C. The medium was changed once during the next 3 days until the cells attained 80% confluence. The cells were then deattached using 0.25% (*w*/*v*) trypsin solution and expanded in a regular growth medium at an initial concentration of 1 × 10^4^ cells/cm^2^. The MSCs were not subjected to chondrogenic induction, and passage-3 cells were employed in the following experiments.

### 2.2. Preparation of Oriented PLGA/ACECM Composite Scaffolds and Cell Seeding

#### 2.2.1. Fabrication of Oriented PLGA/ACECM Composite Scaffolds

Previously, we successfully fabricated oriented PLGA/ACECM composite scaffolds [21, 23]; therefore, we describe the fabrication only briefly. PLGA (70/30) was dissolved in dioxane to 10% (*w*/*v*). The same weight of ACECM was added to this solution to form a slurry. The solid concentration was adjusted to 7% (*w*/*w*) by the addition of dioxane. The mixed slurry was infused into a 4 mm diameter cylindrical mold, which was then inserted vertically into a metal plate and held at −20°C for 30 min. After becoming completely frozen, the mold was held for a further 2 h at −20°C, transferred to a lyophilizer, and lyophilized for 48 h under vacuum. The scaffolds were removed from the molds, and cross-linking proceeded under ultraviolet light at 258 nm for 4 h. The scaffolds were then immersed in 95% (*v*/*v*) alcohol containing 50 mM 1-ethyl-3-(3-dimethylaminopropyl)carbodiimide hydrochloride (EDAC) and 20 mM *N*-hydroxysuccinimide (Sigma-Aldrich, USA) for 24 h at 4°C. Excessive EDAC was washed away repeatedly with phosphate-buffered saline (PBS). The scaffolds were washed in triple-distilled water to remove residual dioxane and then subjected to freeze drying. The scaffolds were finally sterilized by ^60^Co *γ*-irradiation (5 mRad) and stored at 4°C prior to use.

#### 2.2.2. Cell Seeding

MSCs were resuspended in culture medium. Oriented PLGA/ACECM composite scaffolds (4 mm in diameter, 1.8 mm in thickness), sterilized by ethylene oxide, were placed into the wells of a 24-well culture plate. Aliquots (50 *μ*L) of the cell suspension (approximately 2 × 10^6^ cells) were added to completely saturate the scaffolds, which were then incubated under 5% (*v*/*v*) CO_2_ in a humidified atmosphere for 4 h at 37°C to allow for cell adherence, with the addition of 20 *μ*L DMEM (with 10% [*v*/*v*] FBS) to each scaffold every 30 min. Each well of the 24-well culture plate received an additional 1 mL culture medium, and the culture continued for another 3 days.

### 2.3. Surgical Procedure

**Figure 2:**
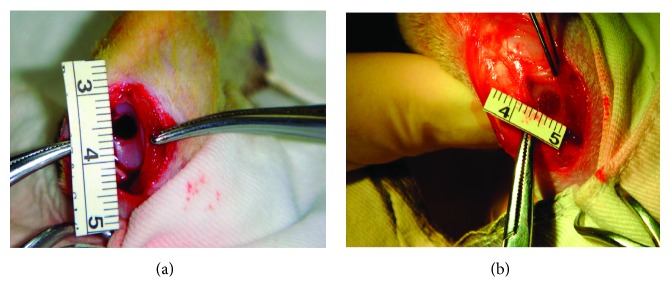
Schematic of the operation. (a) Full-thickness cylindrical defects (3.5 mm in diameter, 1.5 mm in depth) were created on the patellar grooves of the femora of both rabbit legs. (b) MSC-loaded scaffold constructs were implanted into these defects.

The study protocol was approved by the Animal Care and Experimental Committee of our hospital. Adult New Zealand white male rabbits (16–18 weeks) weighing between 2.5 and 4.0 kg were used in this study (*n* = 30). Each rabbit knee joint was opened via a medial parapatellar approach under general anesthesia. Full-thickness cylindrical defects (3.5 mm in diameter, 1.5 mm in depth) were created on the patellar grooves of both femora (reaching the subchondral bone, with minimal bleeding, but not penetrating the bone marrow cavity; we drilled using a corneal trephine; Figure 2).

Surgeries were carried out bilaterally to stifle the joint, and both of the joints received the same treatment. The rabbits were divided randomly into three groups: an MSC-loaded oriented PLGA/ACECM composite-scaffold group (with the MSC/composite scaffold implanted into the cartilage defect), an oriented PLGA/ACECM composite scaffold-alone group (only the scaffold was inserted), and an untreated group. The number of cartilage defects in each group is shown in Table 1. The tissue-engineered constructs were press fitted into the cartilage defects without additional fixation. Postoperatively, all animals were allowed to move freely as soon as they had regained consciousness. To prevent infection, all animals received intramuscular injections of 800,000 units of penicillin on the day of surgery and on each of the next 3 days. The rabbits were euthanized at 12 and 24 weeks postsurgery for evaluation.

### 2.4. Macroscopic Examination and Grading

Rabbits were sacrificed at 12 and 24 weeks postsurgery, and the knee joints were harvested. All evaluations were performed by three independent individuals blinded to the groupings. Repair quality was estimated using the criteria of the International Cartilage Repair Society Macroscopic Evaluation of Cartilage Repair. The scoring system used included rating of the extent of repair, integration with the border zone, and macroscopic appearance (Table 2) [30].

### 2.5. Microcomputed Tomographic Assessment of Subchondral Bone Formation

Microcomputed tomography (micro-CT; GE, USA) was used to quantitatively and qualitatively assess subchondral bone regeneration at 12 and 24 weeks after surgery. A volume of interest was defined in the subchondral bone region of each defect site. Subchondral bone regeneration was estimated in terms of bone volume per tissue volume (BV/TV) and trabecular thickness (Tb.Th).

### 2.6. Histological Examination and Grading

The samples were fixed, decalcified, dehydrated, embedded in paraffin, and sectioned (5 *μ*m thick slices). Next, the slices were stained with hematoxylin and eosin, toluidine blue, and safranin O. Images from each group were recorded via bright-field microscopy (Nikon, Japan). The thickness of each regenerated cartilage layer (from the cartilage surface to the tidemark) was measured and compared with that of the surrounding native cartilage. The extents of toluidine blue and safranin O staining were assessed by counting the integrated optical density (IOD) units per stained area (*μ*m^2^) with the aid of ImageJ software (National Institutes of Health, USA). Grading was performed in a blinded manner by three independent individuals. The extent of repair was evaluated based on the International Cartilage Repair Society grading system. Histological grading involved the evaluation of surface regularity, matrix staining, cell distribution, cell viability, and other items (Table 3) [31].

### 2.7. Immunohistochemical Staining

The expression of collagen type II in regenerated cartilage was examined via immunohistochemical staining at 12 and 24 weeks postimplantation. Collagen type II was stained immunohistologically using a mouse anti-collagen type II polyclonal antibody (Santa Cruz, USA) according to the manufacturer's instructions. In brief, horizontal and vertical sections were blocked with peroxidase-blocking solution for 10 min, followed by three washes with PBS for 5 min each, and each section was then incubated with 50 *μ*L of a solution of anti-collagen type II polyclonal antibody (1 : 50 working dilution) overnight at 4°C. The sections were washed and incubated with biotinylated secondary anti-mouse antibody (Maixin, China), followed by development with diaminobenzidine (Maixin, China). The sections were counterstained with hematoxylin and examined microscopically (Nikon, Japan). Collagen type II immunohistochemical staining was evaluated by calculating the IOD per stained area (*μ*m^2^) with the aid of ImageJ software.

### 2.8. Biomechanical Assessment

The mechanical properties of regenerated cartilage were evaluated by indentation testing using a dedicated apparatus (ElectroForce 3320; Bose, USA), as described previously, at 12 and 24 weeks after surgery. Young's modulus was calculated using the following formula: *E* = *P*(1 − *ν*^2^)/2*awk* (*P*, applied force; *ν*, Poisson's ratio; *a*, indenter radius; *w*, deformation; *k*, area-aspect ratio *a/h* [*h*, cartilage thickness]) [32].

### 2.9. Statistical Analyses

All data were analyzed with the aid of SPSS software (ver. 16; SPSS, Chicago, IL, USA), and the values are expressed as means ± standard deviations. One-way analysis of variance followed by Tukey's post hoc multiple-comparison test was applied to identify significant differences among the groups after assessment of the homogeneities of variance. *P* values < 0.05 were considered to reflect statistical significance.

## 3. Results

### 3.1. Rabbit Health and General Observations on the Joints

#### 3.1.1. Rabbit Health

For 1 week postsurgery, the rabbits reduced their activity levels and appeared to be in a lower mental state than previously; they ate less but exhibited a nearly normal range of joint activity. One rabbit died of a reaction to anesthesia, five died within 1 week due to surgical trauma, three died of diarrhea within 1 month, two died of ear mite infections within 2 months, and one was euthanized because of a joint infection. All rabbits that died before sacrifice were replaced with alternative animals. All remaining rabbits were sacrificed in a timely manner, and the knee joints were obtained.

#### 3.1.2. General Joint Observations prior to Harvest

Before harvesting, the knee skin was complete and was healing well; we observed no swelling or fever and no abnormal secretion. The joint fluids were clear. The synoviae lacked thickening and contracture. The regenerated and surrounding cartilage exhibited no obvious exfoliation or hyperplasia, no patellar dislocation, and no obvious joint instability.

### 3.2. Macroscopic Examination and Grading

**Figure 3:**
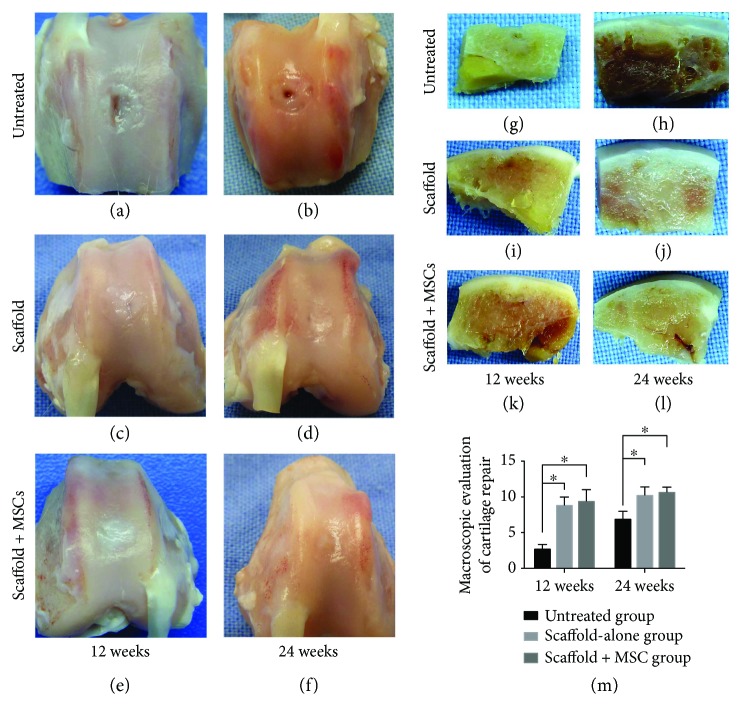
Gross appearance of regenerated cartilage at 12 and 24 weeks postoperation (a–f). Cross-sectional appearance of regenerated cartilage at 12 and 24 weeks postimplantation (g–l). Macroscopic examination and grading of regenerated articular cartilage revealed that the scores for the scaffold-alone and cell-loaded scaffold groups were better than those for the untreated group at 12 and 24 weeks (m). ^∗^*P* < 0.05.

At 12 weeks postsurgery, the cartilage surface in the untreated group was clearly lower than the native cartilage tissue. Moreover, poor integration with surrounding cartilage was evident, and minimal cartilage-like tissue had regenerated (Figure 3(a)). A major osteochondral defect was evident in the cross-sectional view (Figure 3(g)). In the scaffold-alone group, the defect area was almost full of new tissue and the boundary appeared distinct (Figure 3(c)). However, in the cross-sectional view, the central region of the defect was not filled completely with new tissue and regeneration was discontinuous (Figure 3(i)). In the test group, the defect area was filled almost completely with new cartilage tissue, which was comparatively transparent and smooth in appearance. The boundary was distinguishable and exhibited good integration with healthy cartilage (Figure 3(e)). However, subchondral bone had not been reconstituted fully in the central regions, as revealed by the cross-sectional morphology (Figure 3(k)).

At 24 weeks postimplantation, the defective region in the untreated group had increased in size and integration remained poor. Furthermore, the new tissue clearly differed from the native cartilage (Figure 3(b)). In the cross section, the defect region was smaller than that at 12 weeks (Figure 3(h)) In the scaffold-alone group, a cartilage lesional border was still evident, but regions thereof had become vague. Repaired tissue exhibited a comparatively smooth surface and was well integrated with native tissue (Figure 3(d)). In cross-sectional morphological evaluation, the smoothness and continuity were better than those at 12 weeks but the defect boundary remained somewhat uneven (Figure 3(j)). In contrast, in the cell-loaded composite-scaffold group, the defect area was filled completely with regenerated tissue, which was very similar to adjacent healthy cartilage in color and texture. The cartilage defect boundary was vague, and good integration with surrounding native tissue was apparent (Figure 3(f)). In a cross-sectional view, the repaired area did not differ obviously from native cartilage (Figure 3(l)). Macroscopic data and grading of repaired articular cartilage are shown in Figure 3(m). The macroscopic scores of the scaffold-alone group and cell-loaded scaffold group were better than those of the untreated group at 12 and 24 weeks postsurgery (both *P* < 0.05).

### 3.3. Histological Examination and Grading

**Figure 4:**
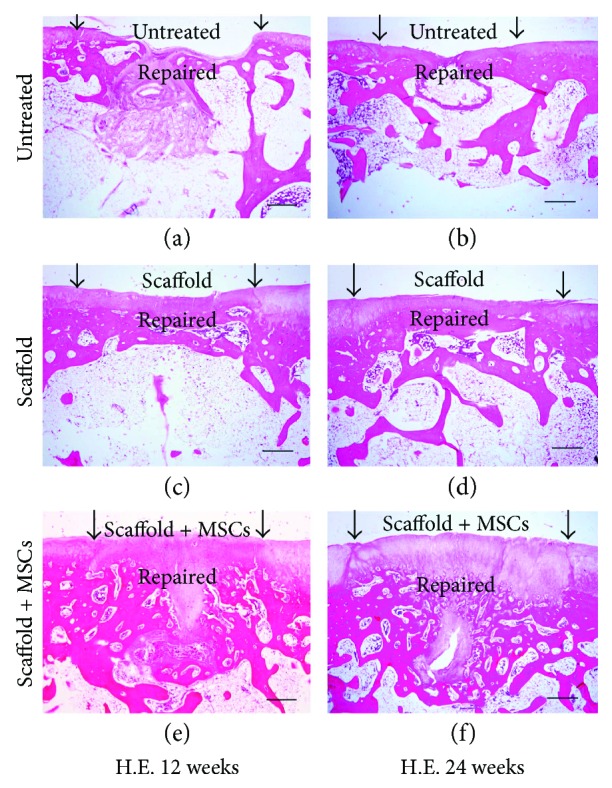
H.E. staining of repaired cartilage at 12 and 24 weeks postimplantation. (a) and (b) At 12 weeks postoperation, the defect area in the untreated group was partially filled with fibrotic tissue. No evident cartilage or subchondral bone regeneration was evident. Moreover, no regeneration was evident at 24 weeks; only fibrous tissue was present. (c) and (d) At 12 weeks postoperation, the defect region was filled with repaired tissue and the boundary exhibited continuous integration with healthy cartilage. The repaired cartilage was clearly thinner than native cartilage. At 24 weeks postimplantation, no further obvious cartilage regeneration was apparent; however, subchondral bone regeneration had progressed further. (e) and (f) At 12 weeks postoperation in the cell-loaded scaffold group, the cartilage defect was filled with regenerated cartilage and subchondral bone and the boundary exhibited continuous integration with healthy cartilage. The thickness of the repaired cartilage was close to that of native cartilage. At 24 weeks postimplantation, cartilage and subchondral bone regeneration were further enhanced and the tissues were similar to native tissues (bars: 50 *μ*m).

**Figure 5:**
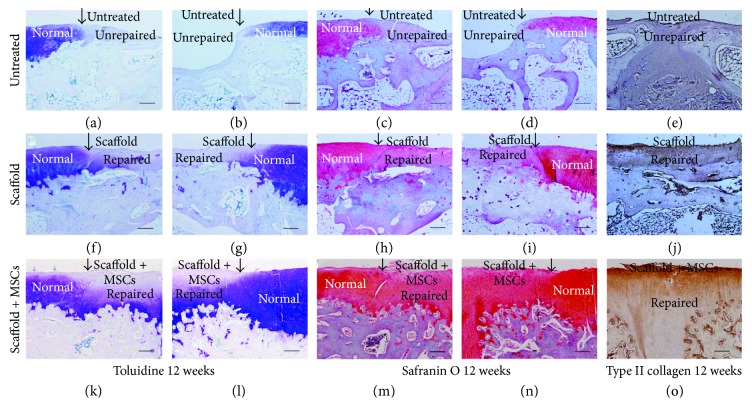
Toluidine blue, safranin O, and immunohistochemical staining of the collagen type II content of repaired cartilage at 12 weeks postimplantation (a–o). (a) and (e) The defect in the untreated group was partially filled with fibrotic tissue but did not stain for cartilage-specific matrix. (f) and (j) The defect region was filled with repaired cartilage, with light staining for cartilage-specific matrix. (k) and (o) The cartilage defect was filled with regenerated cartilage, and the deep regenerated area stained strongly for cartilage-specific matrix (bars: 50 *μ*m).

**Figure 6:**
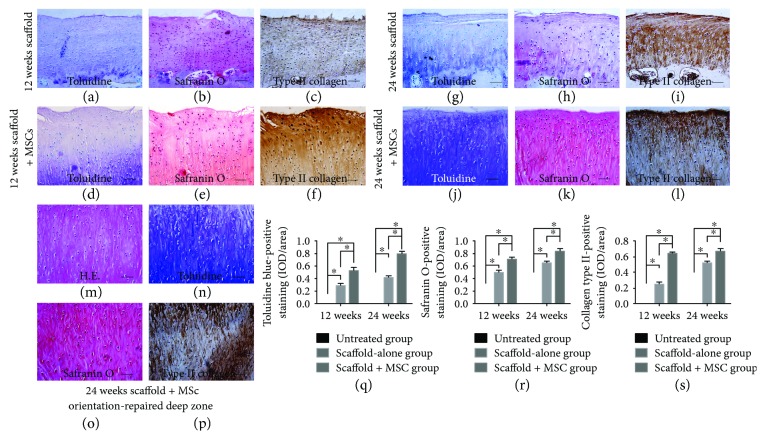
Magnified images of repaired cartilage stained with toluidine blue, safranin O, and immunohistochemical stained for collagen type II at 12 (a–f) and 24 (g–l) weeks postimplantation. The MSC-loaded scaffold group (d–f) exhibited stronger cartilage-specific matrix staining and a more aligned structure than did the scaffold-alone group (a–c) at 12 weeks postimplantation. The cell-loaded scaffold group (j–l) and the scaffold-alone group (g–i) exhibited aligned regeneration at 24 weeks postimplantation, but staining was more intense in the former group than in the latter group. (m–p) Aligned deep-level regeneration of the MSC-loaded scaffold group at 24 weeks postsurgery. (q–s) The IODs per stained area for collagen type II immunoreactivity, and toluidine blue and safranin O staining, at 12 and 24 weeks postimplantation (bars: 10 *μ*m). ^∗^*P* < 0.05.

Macroscopic effects were verified histologically. At 12 weeks postoperation, the defect areas in the untreated group remained concave and no integration with native tissue was evident. No cartilage tissue or subchondral bone regeneration was apparent, except for the development of some fibrotic tissue (Figure 4(a)). In the scaffold-alone group, the defect region was filled with fully repaired tissue and the boundary area exhibited continuous integration with healthy cartilage, although it was slightly uneven (Figure 4(c)). Cartilage-specific matrix staining was uneven (Figures 5(f)–5(j)), and the regenerated tissue was disordered (Figures 6(a)–6(c)). However, in the cell-loaded scaffold group, the cartilage defect region was smooth and continuous and the boundary region was well integrated with adjacent tissue (Figure 4(e)). The staining intensity of the repaired cartilage was stronger than in the scaffold-alone group but weaker than that of native cartilage (Figures 5(k)–5(o)). Most chondrocytes in the deep zone were aligned (Figures 6(d)–6(f)).

**Figure 7:**
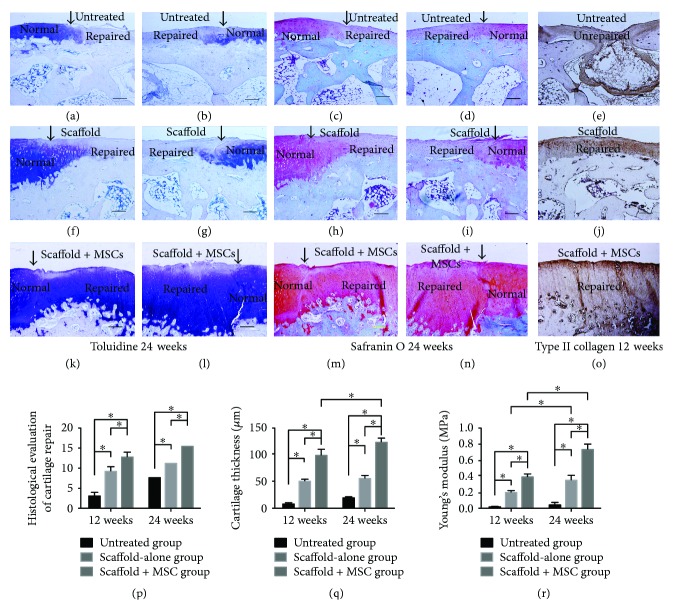
Toluidine blue, safranin O, and immunohistochemical staining of the collagen type II content of repaired cartilage at 24 weeks postimplantation (a–o). (a) and (e) The defect in the untreated group was filled with fibrotic tissue but did not stain for cartilage-specific matrix. (f) and (j) The defect region was filled with repaired cartilage, with light staining for cartilage-specific matrix. (k) and (o) The cartilage defect was filled with regenerated cartilage, and the deep regenerated area stained strongly for cartilage-specific matrix. (p–r) The histological grading, cartilage thickness, and Young's modulus, respectively, of repaired cartilage at 12 and 24 weeks postsurgery (bars: 50 *μ*m). ^∗^*P* < 0.05.

At 24 weeks postsurgery, integration remained poor in the untreated group and few cellular matrices were stained. Most regenerated regions contained only fibrous tissue. Moreover, no subchondral bone regeneration was apparent; again, only fibrous tissue was evident (Figure 4(b)). Conversely, in the scaffold-alone group, the repaired area exhibited a smooth surface and was well integrated with normal cartilage (Figure 4(d)). The repaired tissue was much thinner than native cartilage, but the regenerated cartilage was structurally aligned (Figures 6(g)–6(i)). Matrix staining of regenerated cartilage was less intense than that of native cartilage (Figure 7(f)–7(j)). In the cell-loaded scaffold group, repaired tissue featured a smooth and continuous surface and was well integrated with the surrounding cartilage (Figure 4(f)). Moreover, the thickness of the regenerated cartilage did not differ from that of adjacent cartilage, and the extent of matrix-specific staining was similar to that in healthy cartilage (Figures 7(k)–7(o)). In addition, most chondrocytes in the deep zone were organized in columns (Figures 6(j)–6(p)).

Toluidine blue, safranin O, and immunohistochemical staining for collagen type II at 12 and 24 weeks postsurgery evidenced chondrogenic differentiation in vivo (Figures 5–7). At 12 weeks postoperation, the scaffold-alone group and the cell-loaded scaffold group showed positive staining for collagen type II. The cell-loaded scaffold group exhibited more collagen type II-positive cells than did the scaffold-alone group at 12 and 24 weeks. Moreover, the collagen type II staining level was similar to that of normal cartilage at 24 weeks. However, type II collagen was not expressed in the untreated group. Glycosaminoglycan (GAG) expression in all groups was verified by toluidine blue and safranin O staining at 12 and 24 weeks postsurgery. GAG deposition over time was associated with the expression of collagen type II. The IODs per stained area for collagen type II immunoreactivity, and toluidine blue and safranin O staining, at 12 and 24 weeks showed higher levels of collagen type II and GAG in the cell-loaded scaffold group than in the scaffold-alone and untreated groups (all *P* < 0.05; Figures 6(q)–6(s)). The histological grades of the repaired articular cartilage are shown in Figure 7(p). The scores of the cell-loaded scaffold group were significantly better than those of the other two groups at 12 and 24 weeks postimplantation (all *P* < 0.05).

### 3.4. Microcomputed Tomographic Features of Subchondral Bone

**Figure 8:**
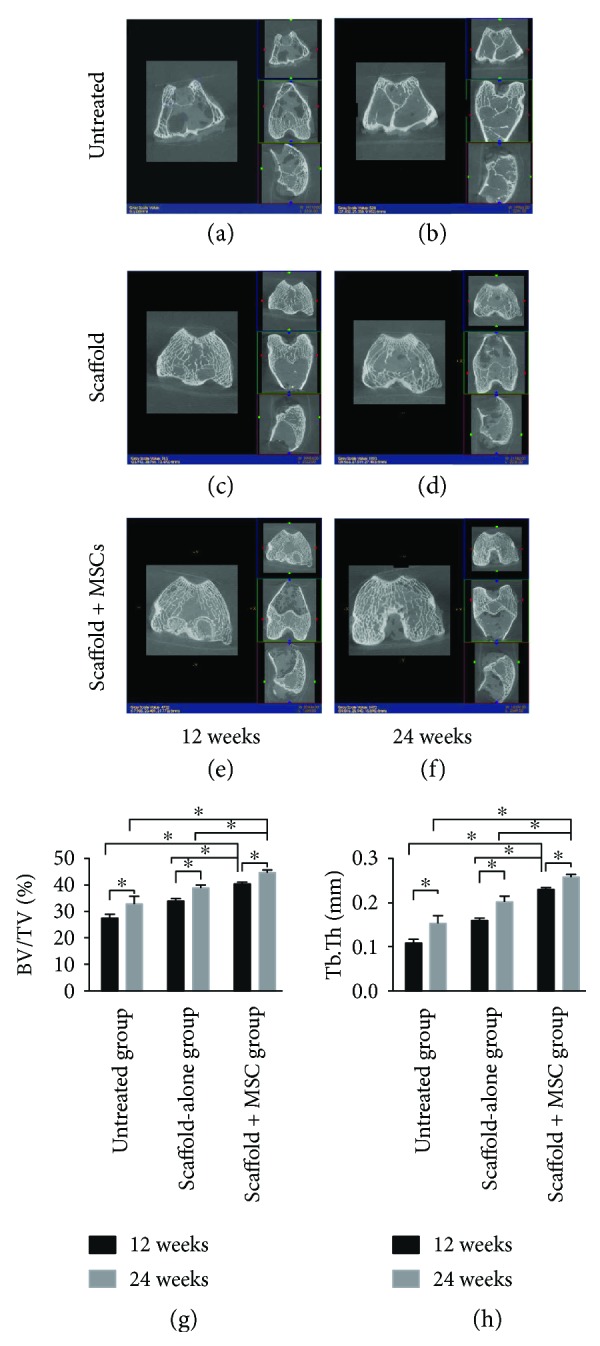
Micro-CT images of regenerated subchondral bone (a–f) at 12 and 24 weeks postimplantation. The bone volume per tissue volume (BV/TV) and trabecular thickness (Tb.Th) data are shown in (g) and (h), respectively. ^∗^*P* < 0.05.

A micro-CT system was used to estimate the quality and quantity of subchondral bone regeneration (Figure 8). Improved levels of subchondral bone formation were evident in all three groups at 12 and 24 weeks after implantation. In terms of the bone volume fraction (the BV/TV), the defect area in the untreated group exhibited inconspicuous signs of regeneration at 12 and 24 weeks postoperation. The scaffold-alone group and the cell-loaded scaffold group exhibited very high levels of subchondral bone repair at 12 and 24 weeks postsurgery (all *P* < 0.05), but these two groups differed significantly at both timepoints (both *P* < 0.05). The BV/TV ratios were significantly higher in the cell-loaded scaffold group than in the scaffold-alone and untreated groups at both timepoints (all *P* < 0.05; Figure 8(g)). In terms of the Tb.Th value, all three groups exhibited trends toward subchondral bone regeneration at 12 and 24 weeks postsurgery (all *P* < 0.05) but significant differences between timepoints were evident (all *P* < 0.05). The Tb.Th values were significantly higher in the cell-loaded scaffold group than in the scaffold-alone and untreated groups at 12 and 24 weeks postsurgery (all *P* < 0.05; Figure 8(h)).

### 3.5. Thickness of Regenerated Cartilage

The thickness of regenerated cartilage was evaluated at 12 and 24 weeks postoperation (Figure 7(q)). It was greatest in the cell-loaded scaffold group at both timepoints (*P* < 0.05). Moreover, from 12 to 24 weeks after implantation, cartilage thickness increased in only the cell-loaded scaffold group (*P* < 0.05) and not in the other two groups (*P* > 0.05).

### 3.6. Biomechanical Assessment of Regenerated Cartilage

The biomechanical properties of the regenerated cartilage were evaluated by calculating Young's modulus at 12 and 24 weeks postoperation (Figure 7(r)). The compressive modulus in the untreated group did not differ significantly between 12 and 24 weeks postoperation (*P* > 0.05). In contrast, the compressive moduli in the scaffold-alone and cell-loaded scaffold groups increased from 12 to 24 weeks postsurgery (*P* < 0.05). Moreover, the compressive modulus was significantly higher in the cell-loaded scaffold group than in the other two groups at both timepoints (*P* < 0.05).

## 4. Discussion

Articular cartilage defects of the knee joint are very common in the general and athletic populations [32]. Cartilage injuries self-heal poorly, triggering OA in the long term [33]. Many surgical interventions, including microfracture and osteochondral autografting or allografting, have been developed to treat cartilage defects [34, 35]. However, no treatment is satisfactory. Many tissue-engineered cartilage approaches have been developed to regenerate cartilage defects. Fabrication of the scaffold is one of the most important steps in such approaches. The scaffold must not only create a suitable microenvironment for tissue regeneration but also be biomechanically competent to resist normal stress forces. We previously developed an oriented PLGA/ACECM composite scaffold, combining the advantages afforded by natural and synthetic polymers. We then combined autologous MSCs with the oriented PLGA/ACECM composite scaffold to restore cartilage defects. Notably, cartilage regenerated completely with a PLGA/ACECM composite scaffold loaded with MSCs was applied. Moreover, the structure and function of the new cartilage and subchondral bone were similar to those of native cartilage. We suggest that our regeneration strategy can be used to repair human cartilage defects.

In the cell-loaded scaffold group, the cartilage defect was repaired almost completely via formation of new cartilage that was similar in appearance to adjacent normal cartilage. Histologically, the regenerated cartilage integrated well with adjacent tissue. Toluidine blue, safranin O, and immunohistochemical staining further confirmed that the cell-loaded scaffold group contained more GAG-expressing and collagen type II-positive cells at 12 and 24 weeks postsurgery. Notably, the newly formed cartilage exhibited the characteristic lacuna-like structure, providing indirect evidence that the cell-loaded scaffold facilitated chondrogenic differentiation in vivo. In addition, most cells in the deeper cartilage defect regions were organized in columns. The cartilage thickness and subchondral bone formation in the cell-loaded scaffold group were similar to those of native tissue. Moreover, from a functional perspective, the regenerated cartilage was biomechanically competent.

**Figure 9:**
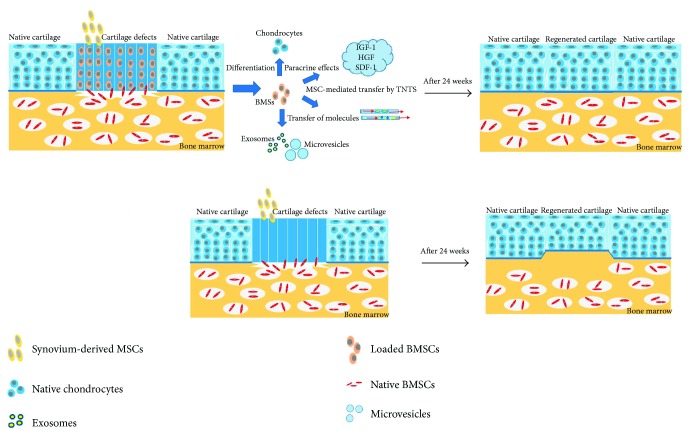
A potential cartilage regeneration mechanism in the MSC-loaded scaffold group. MSCs can differentiate directly into chondrocytes or may secrete growth factors (e.g., insulin-like growth factor (IGF-1) or hepatocyte growth factor (HGF)), rescuing injured tissue. MSCs may also transfer mitochondria via tunneling nanotubes or microvesicles or secrete specific exosomes or microvesicles, influencing surrounding cells.

Repair was not as good in the scaffold-alone group as in the MSC-loaded scaffold group. Some repaired cartilage was evident in the scaffold-alone group at 12 weeks after surgery; however, the newly formed tissue stained only slightly for GAG and type II collagen. Moreover, the cells in this group were arranged randomly. Some studies have shown that ECM degradation products enhance the recruitment and proliferation of multipotential progenitor cells [36]. We speculate that cells in the scaffold-alone group may have been recruited by degraded ECM in vivo. However, in the MSC-loaded scaffold group, MSCs may have additionally promoted tissue regeneration by the following mechanism(s) (Figure 9) [37]. First, MSCs can differentiate directly into native cells; second, the cells may exert paracrine effects via secretion of growth factors or hormones, rescuing injured tissue; third, the cells can transfer mitochondria via tunneling nanotubes or microvesicles; and fourth, the cells may secrete specific exosomes or microvesicles, influencing surrounding cells. Therefore, specific staining and the thickness of repaired cartilage were better in the MSC-loaded scaffold group than in the scaffold-alone group. Autologous MSCs may enhance cartilage and subchondral bone regeneration in these ways. At 24 weeks, the repaired cartilage was more mature than at 12 weeks in the scaffold-alone group, and notably, the regenerated cartilage was aligned. However, specific staining, cartilage thickness, and subchondral bone regeneration were better in the MSC-loaded scaffold group than in the scaffold-alone group. Obviously, the cartilage regeneration evident in the scaffold-alone group and the MSC-loaded scaffold group shows that autologous MSCs can engage in cartilage niche-specific redifferentiation and regeneration when the cells are stimulated by the surrounding ECM in vivo (thus, in the absence of specific growth factors). Previous studies have shown that an aligned scaffold can induce oriented cartilage regeneration [15, 21, 22], as confirmed by the alignment evident in the scaffold-alone group and the MSC-loaded scaffold group in this study. In particular, the better biomechanical capacity in the MSC-loaded scaffold group compared with the scaffold-alone group afforded better functional regeneration.

Stem-cell-loaded scaffold-based strategies are more effective than scaffold-alone strategies [25], as they not only induce aligned cartilage regeneration but also enhance subchondral bone formation. The higher scores for the cell-loaded scaffold group compared with the scaffold-alone group, obtained in blinded macroscopic and histological evaluation, further support this conclusion. However, our study has certain limitations. First, it would have been better to label the added MSCs prior to implantation, to enable determination of the specific roles played by these MSCs in cartilage defect regeneration. Second, a longer observation time would have shown whether regenerated cartilage could maintain good function in the long term and prevent OA development. Third, a large animal (e.g., goat or sheep) model should be used for further evaluation of the efficacy of cartilage regeneration.

## 5. Conclusion

Overall, the results showed that autologous MSCs loaded into an oriented PLGA/ACECM composite scaffold enhanced structure-specific regeneration of hyaline cartilage and subchondral bone in a rabbit model. This approach may become a promising treatment strategy for human cartilage defect repair.

## Figures and Tables

**Table 1:** The number of the cartilage defects in each group over a 24-week-period observation.

Groups	12 weeks postsurgery (*N*)	24 weeks postsurgery (*N*)	Total (*N*)
MSCs loaded with composite scaffolds	5	5	10
Composite scaffolds alone	5	5	10
Untreated group	5	5	10
Total (*N*)	15	15	30

**Table 2:** International Cartilage Repair Society Macroscopic Evaluation of Cartilage Repair.

Cartilage repair assessment ICRS	Points
Degree of defect repair	
In level with surrounding cartilage	4
75% repair of defect depth	3
50% repair of defect depth	2
25% repair of defect depth	1
0% repair of defect depth	0
Integration to border zone	
Complete integration with surrounding cartilage	4
Demarcating border < 1 mm	3
3/4th of graft integrated, 1/4th with a notable border > 1 mm width	2
1/2 of graft integrated with surrounding cartilage, 1/2 with a notable border > 1 mm	1
From no contact to 1/4th of graft integrated with surrounding cartilage	0
Macroscopic appearance	
Intact smooth surface	4
Fibrillated surface	3
Small, scattered fissures or cracks	2
Several small or few but large fissures	1
Total degeneration of grafted area	0
Overall repair assessment	
Grade I: normal	12
Grade II: nearly normal	11–8
Grade III: abnormal	7–4
Grade IV: severely abnormal	3–1

ICRS: International Cartilage Repair Society.

**Table 3:** International Cartilage Repair Society Visual Histological Assessment Scale for Cartilage Repair.

Feature	Points
I. Surface	
Smooth/continuous	3
Discontinuities/irregularities	0
II. Matrix	
Hyaline	3
Mixture: hyaline/fibrocartilage	2
Fibrocartilage	1
Fibrous tissue	0
III. Cell distribution	
Columnar	3
Mixed/columnar clusters	2
Clusters	1
Individual cells/disorganized	0
IV. Cell population viability	
Predominantly viable	3
Partially viable	1
<10% viable	0
V. Subchondral bone	
Normal	3
Increased remodeling	2
Bone necrosis/granulation tissue	1
Detached/fracture/callus at base	0
VI. Cartilage mineralization (calcified cartilage)	
Normal	3
Abnormal/inappropriate location	0
